# In Vitro Analysis of Cytotoxic Activities of *Monotheca buxifolia* Targeting *WNT*/*β-Catenin* Genes in Breast Cancer Cells

**DOI:** 10.3390/plants12051147

**Published:** 2023-03-02

**Authors:** Ambreen Sher, Sobia Tabassum, Heather Mann Wallace, Asifullah Khan, Asad Mustafa Karim, Sarah Gul, Se Chan Kang

**Affiliations:** 1 Department of Biological Sciences, Faculty of Basic and Applied Sciences, International Islamic University, Islamabad 44000, Pakistan; 2 Institute of Medical Sciences, University of Aberdeen, Aberdeen AB24 3FX, UK; 3 Department of Computer and Information Sciences, Pakistan Institute of Engineering and Applied Sciences (PIEAS), Islamabad 44000, Pakistan; 4 Department of Oriental Medicine Biotechnology, College of Life Sciences, Kyung Hee University, Yongin-si 17104, Republic of Korea

**Keywords:** *Monotheca buxifolia*, natural products, cytotoxicity, anticancer, *WNT* signaling

## Abstract

Breast cancer (BC) is known to be the most common malignancy among women throughout the world. Plant-derived natural products have been recognized as a great source of anticancer drugs. In this study, the efficacy and anticancer potential of the methanolic extract of *Monotheca buxifolia* leaves using human breast cancer cells targeting *WNT*/*β-catenin* signaling was evaluated. We used methanolic and other (chloroform, ethyl acetate, butanol, and aqueous) extracts to discover their potential cytotoxicity on breast cancer cells (MCF-7). Among these, the methanol showed significant activity in the inhibition of the proliferation of cancer cells because of the presence of bioactive compounds, including phenols and flavonoids, detected by a Fourier transform infrared spectrophotometer and by gas chromatography mass spectrometry. The cytotoxic effect of the plant extract on the MCF-7 cells was examined by MTT and acid phosphatase assays. Real-time PCR analysis was performed to measure the mRNA expression of *WNT-3a* and *β-catenin*, along with *Caspase-1,-3,-7,* and *-9* in MCF-7 cells. The IC50 value of the extract was found to be 232 μg/mL and 173 μg/mL in the MTT and acid phosphatase assays, respectively. Dose selection (100 and 300 μg/mL) was performed for real-time PCR, Annexin V/PI analysis, and Western blotting using Doxorubicin as a positive control. The extract at 100 μg/mL significantly upregulated caspases and downregulated the *WNT-3a* and *β-catenin* gene in MCF-7 cells. Western blot analysis further confirmed the dysregulations of the *WNT* signaling component (*** *p<* 0.0001). The results showed an increase in the number of dead cells in methanolic extract-treated cells in the Annexin V/PI analysis. Our study concludes that *M. buxifolia* may serve as an effective anticancer mediator through gene modulation that targets *WNT*/*β-catenin* signaling, and it can be further characterized using more powerful experimental and computational tools.

## 1. Introduction

It is recognized by professionals that malignancy is the consequence of irregular and/or inheritable genetic mutations in germinal or somatic cells [1]. It is intimidating how malignancy spans genetics, cell and tissue biology, pathology, and therapeutic response. Ever more powerful experimental and computational tools and technologies are producing a tsunami of “big data” on the various symptoms of diseases, including cancer. In an effort to better understand the mechanisms of cancer genesis and malignant progression in order to apply this knowledge to cancer care, the preliminary new dimensions offered in this perspective may be helpful. This intricacy is being reduced to a more logical science with the help of the integrative idea reflected in the hallmarks of cancer [2]. In 2022, the United States was expected to have 1,918,030 new cancer cases and 609,360 cancer deaths [3]. Breast cancer is one of the most dangerous and frequently occurring cancers in the world. It is the most prevalent cancer in women and accounts for 30% of female cancers [3]. Estimation studies are very much alarming for breast cancer prevalence in Pakistan, which has seen an estimated 60.7% increase in its incidence [4]. Approximately 10% of women with BC have a family history of the illness.

Cancer is the presence of unstable physiological signaling pathways due to mutation at genetic or post genetic levels that are otherwise under firm control [5]. Numerous molecular mechanisms and signaling pathways relating to BC proliferation, metabolism, survival, and mobility have recently been identified by research, and these pathways ultimately contribute to the disease’s resistance to novel targeted therapies. Among all signaling pathways, *WNT/β catenin* is the most conserved signaling pathway, and is mostly involved in the growth and development processes in all animal species; hence, any disruption in the process can lead to abnormality at the cellular level [6]. In a controlled manner, when a *WNT* ligand binds to its frizzled (Fz) receptor, it activates a disheveled (Dvl) protein through its phosphorylation, which binds to a glycogen synthase kinase 3-β (GSK3-β), an inhibitor of *β-catenin*. Thus, GSK3-β is no longer available to block *β-catenin*, which is now free in the cytoplasm to perform its role. One of the pathways that *β-catenin* adopts is that it moves to the nucleus and interacts with various transcription factors, such as T-cell factor (TCF) or lymphoid enhancer factor (LEF), to transcribe its targeted genes. The well-studied targets of *β-catenin* are *c-myc*, *cyclin D1*, *c-jun* and *CDH-1*, all of which are involved in the proliferation of cells; thus, their overexpression can cause abnormalities [7]. Many natural, synthetic, and semisynthetic compounds are known to control the proliferation of tumors targeting *WNT/β-catenin* signaling [8]. These inhibitor compounds can be used in controlling *WNT* signaling by acting as antagonists to block the overactivation of the cascade involved in proliferation. Nowadays, the most frequent treatment for cancer, including breast cancer, is chemotherapy, which controls the excessive proliferation of cancerous cells. However, it also destroys the normal cells, and causes severe side effects [9]. Thus, there is a dire need for therapeutics that would have lesser side effects and that are cost-effective when compared to chemotherapy.

Breast cancer treatments, such as chemotherapy, surgery, and others, have been shown to reduce cancer-related fatalities. Current medications or extra ways to combat chemotherapy-induced adverse effects are frequently ineffective, frequently fail to address potential long-term consequences, and may even cause further side effects that contribute to patient misery. As a result, extensive research is now being conducted to find better chemotherapy medications derived from naturally occurring substances that can reduce or halt the process of carcinogenesis. The rate of apoptosis has a significant impact on the life span of both cancer cells and normal cells. As a result, regulating apoptosis may be useful in cancer management, therapy, or prevention. Natural products, in particular, provide such templates [10]. Hence, it is imperative that apoptotic inducers be screened from plants, either in the form of compounds or crude extracts from them. Scientific reports regarding the anticancer therapeutic potential of *Monotheca buxifolia* (*M. buxifolia*) are lacking. Hence, in an attempt to provide scientific evidence, we evaluated the cytotoxic and apoptotic induction potential of *M. buxifolia* leaves extract in human breast cancer cell lines.

*M. buxifolia* is a broad-leaved perennial tree belonging to the Sapotaceae family. This species is also distributed in the alpine areas in Northern Pakistan, Northern Oman, and in southeast Saudi Arabia. In folk medicine, its fruit is used for its laxative, antipyretic, hematinic, vermicidal, and purgative properties, as well as for the management of gastro-urinary disorders [11]. The leaves of this plant comprise flavonoids, anthraquinones, cardiac glycosides, terpenoids, tannins, saponins, reducing sugars, and poly-phenolic compounds [12]. This study was designed to evaluate the in vitro cytotoxic efficiency of a methanol extract of *M. buxifolia* leaves (MMBL) with regard to breast cancer cell lines. So far, no work has been reported for *M. buxifolia* targeting *WNT/β-catenin* signaling in different cancers.

## 2. Results

### 2.1. Percentage Yield

The percentage yield of MMBLwas 20 g per 100 g of dry powder using the maceration method. The percentage yield in chloroform, n-hexane, and ethyl acetate was 3.5 g, 1.5 g, 1 g, and 4 g of butanol extract, respectively. Hence, MMBL was used for further experimentation due to its maximum yield.

### 2.2. Total Phenolic/Total Flavonoid Contents

There are many active compounds working as reducing agents, but phenols and flavonoids have gained special importance due to their scavenging capacity and some other biological activities. The results showed that the MMBL possessed 60.5 ± 2.8 mg of gallic acid/g of leaf extract and 28.26 ± 2.4 mg of rutin/g of leaf extract (Figure S1).

### 2.3. Fourier Transform Infrared Spectrophotometer (FTIR) and Gas Chromatography Mass Spectrometry (GCMS) Analyses

The presence of secondary metabolites was confirmed by a FTIR. The FTIR spectroscopic studies revealed different characteristic peak values with various functional compounds in the extracts. The peak at 3020 cm^−1^ revealed the presence of amides, the peak at 1750 cm^−1^ revealed the presence of ketones, and, similarly, the peak at 1540 cm^−1^ revealed the presence of aldehydes. The peak at 932 cm^−1^ indicated the presence of primary alcohol (Supplementary Figures S2 and S3). The GCMS analysis found 93 bioactive compounds, some of which have been reported for biological activity. Table 1 shows their retention time (min.), abundance (%), and molecular formula mass (a.m.u.), as well as their chemical structures. The most abundant molecules were 9,12-octadecadienoic acid (Z, Z) (% abundance 19.54), sitosterol (% abundance 15.95), n-hexadecanoic acid (% abundance 6.68), and 1,2 benzenedicarboxylic acid (% abundance 6.17) (Table 1).

### 2.4. Radical Scavenging Assays

The EC50 value in the DPPH assay was 0.31 ± 0.02 mg/mL. The phosphomolybdenum assay was expressed in ascorbic acid equivalency—AEE (mg of ascorbic acid equivalent/g of extract). The MMBL indicated 56.4 ± 2.06 mg of ascorbic acid equivalent/g extract. The results showed a decrease in concentration with each serial dilution.

### 2.5. Cell Viability Assays

**Figure 1:**
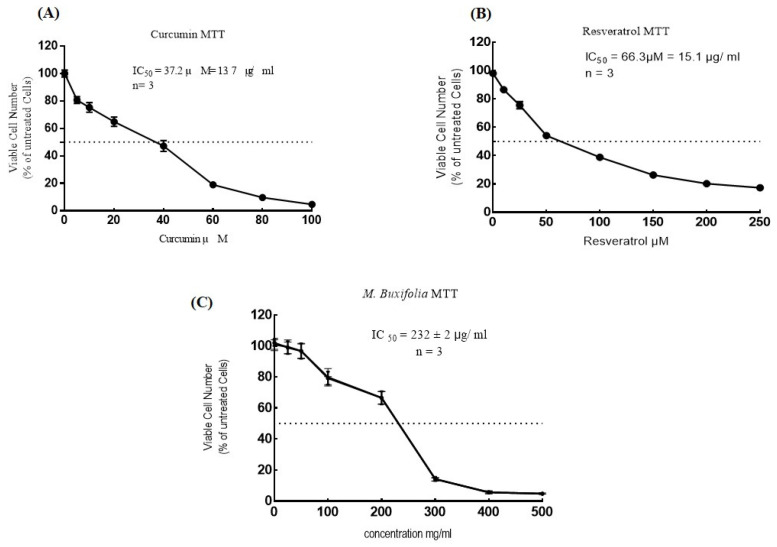
Analysis of percentage of MCF-7 cell death by MTT assay at different doses of (**A**) Curcumin, (**B**) Resveratrol, and (**C**) MMBL after 48 h of incubation. Each value represents a mean ± SD (*n* = 3). *MMBL: methanolic extract of M. buxifolia leaves;* MTT: (3-(4,5-dimethylthazolk-2-yl)-2,5-diphenyl tetrazolium bromide) assay; APT: acid phosphatase assay.

**Figure 2:**
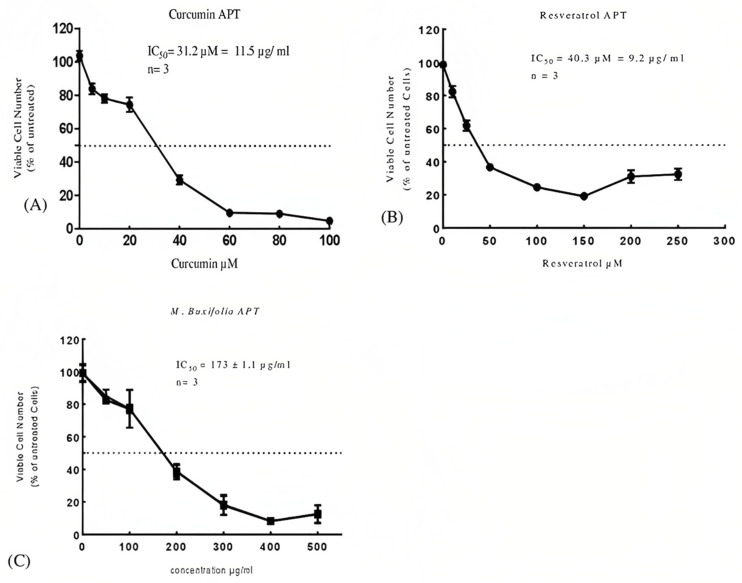
Analysis of percentage of MCF-7 cell death by acid phosphatase assay at different doses of (**A**) Curcumin, (**B**) Resveratrol, and (**C**) MMBL after 48h of incubation. Each value represents a mean ± SD (*n* = 3). *M. buxifolia* = MMBL, MTT = (3-(4,5-dimethylthazolk-2-yl)-2,5-diphenyl tetrazolium bromide) assay, APT = acid phosphatase assay.

The effect of the MMBL on the viability of MCF-7 breast cancer cell lines was assessed by an MTT and acid phosphatase assay (APT). The percentage inhibition results were estimated after incubating the cells with anticancer agents for 48 h. The dosing range (0 to 500 μg/mL) was selected for cytotoxicity analysis. The IC50 value of the MMBL was found to be 232 μg/mL for the MTT and 173 μg/mL for the acid phosphatase assay. In this study, we used curcumin and resveratrol as standard natural drugs to compare their effects with the MMBL (Figure 1 and Figure 2, Table 2).

### 2.6. Real-Time Fold Expression of Targeted Genes

#### 2.6.1. Upregulation of *Caspase-1, -3, -7,* and *-9*

The present study showed that expression of *Casp-1, -3, -7, and -9* was increased in MCF-7 breast cancer cell lines upon treatment with the MMBL, using 100 μg/mL and 300 μg/mL, compared to non-treated MCF-7 cells. The ∆∆Ct mean of the

**Figure 3:**
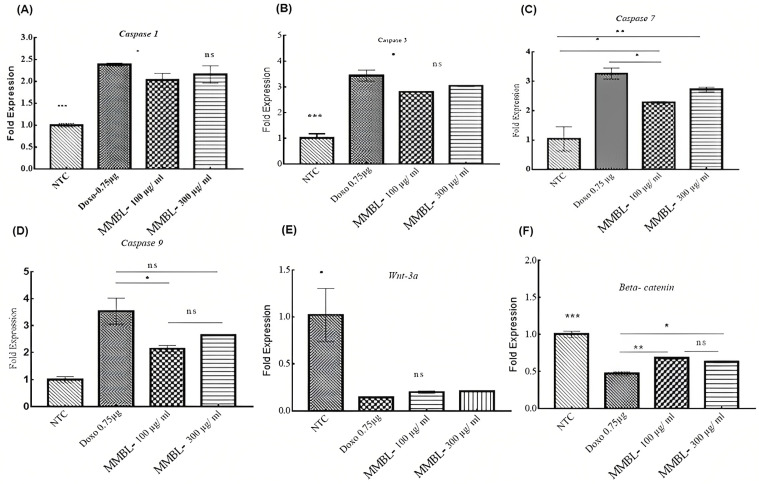
Comparison of change in the expression of *Casp-1, -3, -7*, and *-9* genes expressed as target gene/GAPDH in MCF-7 cells after 24 h exposure with MMBL. NTC: non-treated cells; Doxo: Doxorubicin; a positive control, MMBL: methanolic extract of *M. buxifolia* leaves. (**A**) *p* < 0.0002 = *** treated cells vs. non-treated cells. (**B**) *p* < 0.001 = *** treated cells vs. non-treated cells. (**C**) *p* < 0.001 = *** treated cells vs. non-treated cells. (**D**) *p* < 0.001 = *** treated cells vs. non-treated cells. (**E**) *p* < 0.0092 = ** treated cells vs. non-treated cells. (**F**) *p* < 0.0002 = *** treated cells vs. non-treated cells. All the experiments were repeated twice with triplicate. Where * denotes *p* < 0.05 in treated cells vs. non-treated cells.

*Casp-1* gene in treated MCF-7 cells by the Tukey’s test analysis between the two different doses of MMBL showed no statistical difference between the two doses (*p* < 0.75) compared to the *GAPDH*. Similarly, when comparing the treated cells with the positive control Doxorubicin 0.75 μg, it was also statistically non-significant (*p* < 0.15 at 100 μg/mL and *p* < 0.41 at 300 μg/mL). In contrast to the non-treated MCF-7 cells that were not undergoing apoptosis, the ∆∆Ct mean of the *Casp-1* gene was much lower compared to both doses of the MMBL and to Doxorubicin; it was statistically highly significant (*p* < 0.001). By using centered on one-way analysis of variance, our results showed a statistical difference among the fold expression of the *Casp-1* gene in treated versus non-treated cells (*p* < 0.001) that indicated the upregulation of *Casp-1* in MCF-7 cells upon treatment with the MMBL at two different doses (Figure 3A), as is shown in Table 3.

The ∆∆Ct mean of the *Casp-3* gene resulting from the multiple comparison by Tukey’s test analysis between 100 μg/mL and 300 μg/mL doses of MMBL, as well as 0.75 μg of the positive control Doxorubicin showed no significant statistical difference (*p* < 0.44). Similarly, when comparing the treated cells, the fold expression was 3.44 ± 0.22, which was statistically non-significant in comparison with 300 μg/mL of MMBL (*p* < 0.14), but comparing its expression level with 100 μg/mL was found to be statistically significant (*p* < 0.03). In contrast to the non-treated cells, the ∆∆Ct mean of the *Casp-3* gene was much lower when compared to both doses of the MMBL and to Doxorubicin, which were found to be statistically significant (*p* < 0.001). Based on one-way analysis of variance, our results showed a statistical difference among the fold expression of the *Casp-3* gene in treated versus non-treated cells (*p* < 0.001), which indicated upregulation of the *Casp-3* in MCF-7 cells upon treatment with the MMBL at two different doses (Figure 3B, Table 3).

Similarly, the mean relative fold expression of the *Casp-7* gene from the multiple comparison by Tukey’s test analysis between the two different doses of MMBL showed no statistical difference (*p* < 0.33) when compared to the *GAPDH* gene. By comparing the treated cells with the positive control, for Doxorubicin (0.75 μg), the fold expression was statistically non-significant in comparison with 300 μg/mL (*p* < 0.23), but comparing its expression level with 100 μg/mL it was found to be statistically significant (*p* < 0.04). In contrast to the non-treated MCF-7 cells, the ∆∆Ct mean of the *Casp-7* gene was much lower compared to both doses of the MMBL and to Doxorubicin, and it was found to be strongly significant statistically (*p* < 0.002). By using centered on one-way analysis of variance, our results showed a statistical difference among the fold expression of the *Casp-7* gene in treated versus non-treated cells (*p* < 0.002), which indicated the upregulation of *Casp-7* in MCF-7 cells upon treatment with the MMBL at two different doses (Figure 3C and Supplementary Table S1).

The mean relative fold expression of the *Casp-9* gene compared to the *GAPDH* at the two different doses of MMBL showed no statistical difference (*p* < 0.34). Likewise, when comparing the treated cells with the positive control Doxorubicin (0.75 μg), the fold expression was statistically non-significant in comparison with 300 μg/mL (*p* < 0.08), but comparing its expression level with 100 μg/mL was found to be statistically significant (*p* < 0.01). By using centered on one-way analysis of variance, the results showed a statistical difference among the fold expression of the *Casp-9* gene in treated versus non-treated cells (*p* < 0.001), which indicated the upregulation of *Casp-9* in MCF-7 cells upon treatment with *M. buxifolia* plant extract at two different doses. The MMBL inhibited anti-apoptosis in MCF-7 cells by directing caspase activation. ((Figure 3D) and Supplementary Table S1).

#### 2.6.2. Downregulation of *WNT-3a* and *β-Catenin*

In our study, the expression of *WNT-3a* and *β-catenin* genes was found to be decreased in MCF-7 cells upon treatment with the MMBL (100 μg/mL and 300 μg/mL) when compared to non-treated MCF-7 cells. The mean relative fold expression of *WNT-3a* and *β-catenin* genes compared to the *GAPDH* by Tukey’s test analysis between the two different doses of MMBL and Doxorubicin showed no statistical difference. In contrast to the non-treated MCF-7 cells, the ∆∆Ct mean of the *WNT-3a* gene was much higher compared to both doses of the MMBL and to Doxorubicin, and it was statistically significant (*p* < 0.01). Based on one-way analysis of variance, our results displayed a statistical difference among the fold expression of the *WNT-3a* gene in treated versus non-treated cells (*p* < 0.0092), which indicated downregulation of the *WNT-3a* and *β-catenin* genes in MCF-7 cells upon treatment with the MMBL at 100 μg/mL and 300 μg/mL (Figure 3E,F and Supplementary Table S2). These results indicate that MMBL could serve as an effective anticancer mediator against breast cancer cells through dynamic gene modulation in cancer signaling.

### 2.7. MMBL-Induced Apoptosis in MCF-7 Cells

Phosphatidylserine externalization on the cell membrane is a hallmark of apoptotic cells. Annexin V is a recombinant protein with a strong affinity for this externalized moiety that can detect apoptosis. The Annexin V FITC propidium iodide dual labelling was used to assess apoptotic cell death. To determine whether apoptotic cell death was responsible for decreases in cell viability, we performed Western blot and flow cytometry analysis with Annexin V and PI staining. MCF-7 cells were treated with Doxorubicin (0.75 μg/mL) and MMBL (100 μg/mL). The results showed that the apoptosis rates (Annexin V+ and PI-) in the MMBL-treated cells were significantly increased in the MCF-7 cell lines compared with the non-treated cells (*p* < 0.0001). These results suggest that the MMBL had significantly inhibited cancer growth primarily through inducing cancer cell apoptosis.

**Figure 4:**
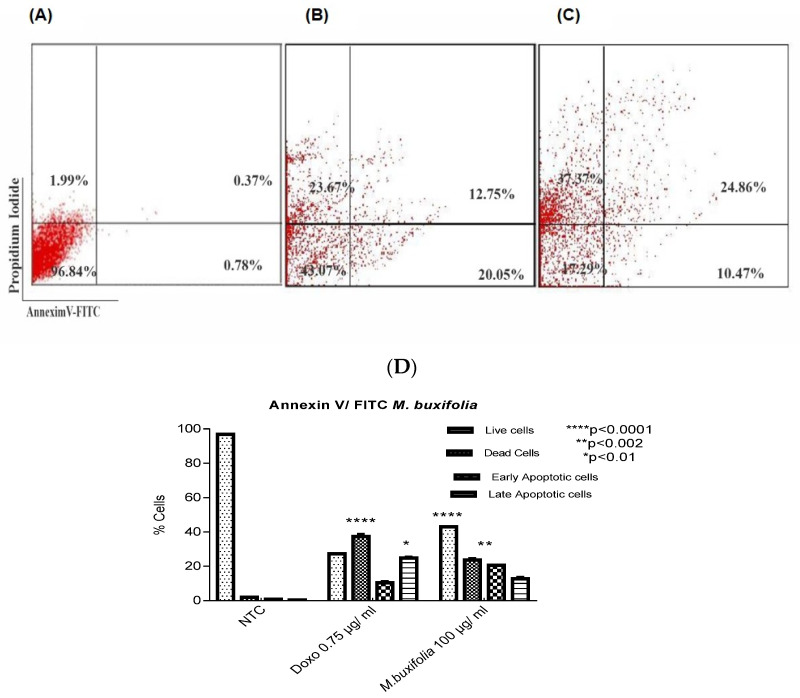
Representative flow cytometric dot plots representing individuals showing values close to mean values in all three groups. Apoptosis assay for MCF-7 cells isolated from NTC (non-treated) (**A**), Doxo = Doxorubicin-treated cells at 0.75 μg/mL (**B**) and MMBL-treated at 300 μg/mL (**C**). Four quadrants are shown i.e., lower left (LL) representing viable cells, upper left (UL) representing dead cells, lower right (LR) representing early apoptotic cells and upper right (UR) representing late apoptotic cells. (**D**). Graph showing percentage population of different cell types in NTC = non-treated cells, Doxorubicin-treated group, and MMBL-treated group. A highly significant (*p* < 0.0001) difference was observed between live and dead cells in non-treated and treated group. The results showed that the MMBL-treated cells significantly increased apoptotic cells populations compared to untreated control cells. Significant data points are represented with asterisk mark.

The apoptotic assay data was represented as mean ± SD. The percentage viability of MCF-7 non-treated cells, MMBL-treated cells, and Doxorubicin-treated cells measured as 96.84 ± 0.40 (Figure 4A, Quad LL), 43.07 ± 0.00 (Figure 4B, Quad LL), and 27.29 ± 0.02 (Figure 4C, Quad LL), respectively. The percentage viability of MCF-7 cells was significantly higher in untreated cells compared to treated cells i.e., the *p* < 0.0001 between the groups (non-treated vs. treated and Doxorubicin-treated). Assessment of percentage of the apoptosis in MCF-7 cells of treated and non-treated cells was further subdivided into dead (Annexin V FITC−/PI+) cells, early apoptotic (Annexin V FTIC+/PI−), and late apoptotic (Annexin V FITC+/PI+) cells. The percentages of dead MCF-7 cells in non-treated, MMBL-treated, and Doxorubicin-treated cells were measured as 1.99 ± 0.20 (Figure 4A, Quad UL), 23.67 ± 1.06 (Figure 4B, Quad UL), and 37.37 ± 1.4 (Figure 4C, Quad UL). Untreated MCF-7 cells depicted statistically significant data points (*p* < 0.0001). The percentage of dead MCF-7 cells in non-treated, MMBL-treated, and Doxorubicin-treated conditions was measured as 0.78 ± 0.30 (Figure 4A, Quad LL), 20.05 ± 0.00 (Figure 4B, Quad LL), and 10.47 ± 0.70 (Figure 4C, Quad UL). Data showed similar statistical significance i.e., a *p* < 0.0001 between the groups (treated and untreated). The percentage of dead MCF-7 cells in non-treated, MMBL-treated, and Doxorubicin-treated conditions was measured as 0.37 ± 0.10 (Figure 4A, Quad UR), 12.75 ± 1.06 (Figure 4B, Quad UR), and 24.86 ± 0.70 (Figure 4C, Quad UR), respectively. Data showed a similar statistical significance, i.e., a *p* < 0.0001 (Figure 4D).

The overall results showed increased cell death, apoptosis, and decreased viability of the treated MCF-7 cells. A higher percentage of cells in the early and late apoptotic stages was found in the treated cells, and it was lower in non-treated cells.

### 2.8. Western Blotting

**Figure 5:**
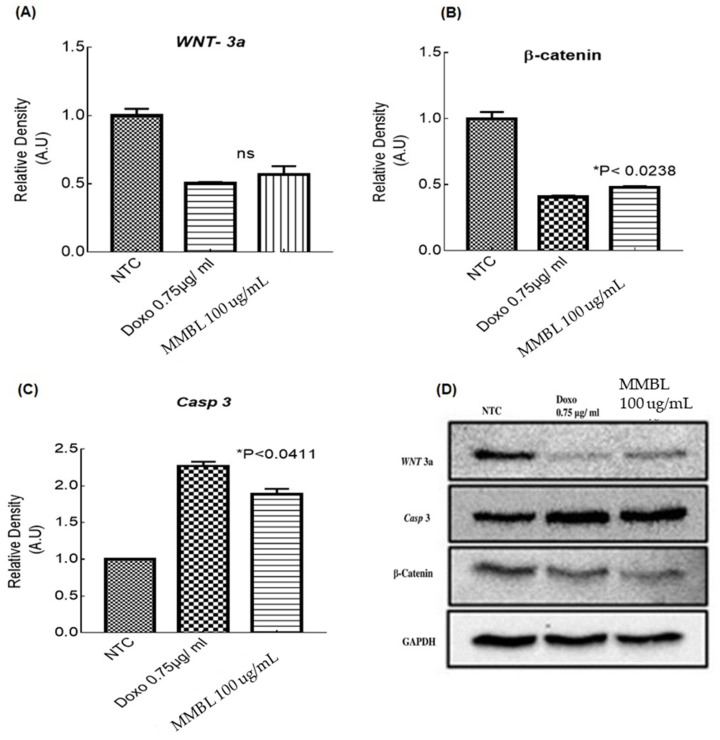
Effect of MMBL on *WNT*/*β-catenin* signaling by Western blot analysis. MCF-7 was treated with MMBL 100 μg/ml and then Western blot analysis was performed. One-way ANOVA with Tukey’s multiple comparisons test between MMBL 100 μg/ml and Doxorubicin treated cells showed (**A**) *WNT-3a* (ns: no significant difference), (**B**) *β-catenin* (* *p* < 0.00238) and (**C**) *Caspase 3* (* *p* < 0.041) MCF-7 cells. GAPDH immunoblotting served as the loading control. (**D**) Western blot showing the comparative concentration of targeted genes.

Protein extracts from breast cancer cells were prepared to determine whether the cytotoxic effect of 100 μg/mL of MMBL in MCF-7 cells was mediated by the modulation of *WNT*/*β-catenin*-signaling components. Western blot analysis revealed that the MMBL 100 μg/mL treatment significantly reduced *WNT-3a* and *β-catenin* expression, and Doxorubicin was used as a control. *Caspase 3* expression was observed to be higher (*p* < 0.0003), as is shown in Figure 5, and *GAPDH* was used as a reference (Figure 5).

## 3. Discussion

*M. buxifolia*, commonly known as gurguri, is a local fruit found in northern areas of Pakistan. It is used by locals to treat various illnesses. After many years of research, it has been shown to have a high concentration of phenols and flavonoids, which gives this plant unique scavenging properties against ROS [8]. To test the cytotoxic potential of this plant, many preliminary tests were carried out confirming the antioxidant potential of this plant along with a targeted study of *WNT* signaling in MCF-7 cell lines. The plant metabolites serve as antioxidants that can chelate free radicals, thus protecting cellular organelles from disruptions such as lipid, protein, and DNA damage. The antioxidants provide hydrogen ions that halt the free radical chain and close the ROS activity. The oxidative chain reaction, from its initiation to progression, can be prevented by primary metabolites that neutralize free radicals, whereas secondary metabolites suppress the process of ROS formation to thus control further oxidative damage [23]. Our study has shown that the MMBL has strong antioxidant potential, with an effective EC50 value of 0.31 ± 0.02 mg/mL and 0.1 ± 0.01 mg/mL found in the DPPH assay. Similarly, the phosphomolybdate assay found 56.4 ± 2.06 AEE mg/g extract. The total phenolic content and total flavonoid content ranges were 60.5 ± 2.8 GAE mg/g extract and 28.26 ± 2.4 RE mg/g extract, respectively, which is similar with results from previous studies [23,24]. The FTIR peaks shown by the MMBL were found to be closer to reported studies, which ensure the strong antioxidant properties of the plant [10] and, thus, ensure the presence of functional groups of active biomolecules. A total of 93 bioactive compounds were identified through GCMS analysis, where 9,12-cctadecadienoic acid, sitosterol, n-hexadecanoic acid, and 1,2 benzenedicarboxylic acid were found in maximum abundance, and they are known to have chemoprotective effects as reported by [16,19,21,22]. Cytotoxicity analysis is the powerful basic tool to analyze the antioxidant activity of test samples in vitro [25]. In this study, we used MMBL for cytotoxicity analysis along with standard drugs and found comparable results. The MTT assay for the MMBL found an IC50 value of 232 μg/mL, and comparable results have been stated by [26]. For the acid phosphatase assay, the MMBL revealed an IC50 value of 173 μg/mL, which has not been reported previously, because no such assay has been performed using MMBL. Real-time PCR to analyze the fold expression of *Caspase-1, -3, -7,* and *-9*, as well as the *WNT-3a* gene and *β-catenin* gene, was performed using MMBL at 100 μg/mL and 300 μg/mL, and this revealed the upregulation of caspases and the downregulation of the *WNT-3a* gene and *β-catenin* gene.

Our results have also been validated by similar reported studies. Wang et al., (2021) have shown that *Casp-1* is involved in pyroptosis, which is a *Caspase-1*-dependent cell death that is activated by a range of microbial infections and cancers [27]. Pang et al., (2021) worked on Angelicakeiskei, a Japanese perennial plant. They extracted Xanthoangelol (XAG), to study its effect on *Casp-1*-dependent pyroptosis and found a significant increase in *Casp-1* activity in *Hep-G 2* cell lines in a drug-dependent fashion [28]. Al-Oqail et al., (2019) worked on the cotton silk extract of *Zea mays* by using a different range of concentrations for 24 h and found a significant upregulation in the fold expression of *Casp-3* and *-9*, which indicated increased apoptosis in a dose-dependent manner (*p* < 0.05) using real-time PCR [29]. Yaoxian et al., (2013) worked on an anthraquinone derivative, Emodin, which was separated from a Chinese herb, *Rheum palmatum* L. The *Casp-9* and *-3*, assessed at the mRNA level using real-time PCR, after treatment with Emodin, were significantly (*p* < 0.05) increased in a dose-dependent manner by persuading apoptosis over mitochondrial and death receptor pathways [30]. Srinivas et al., (2003) also worked on Emodin and found an increase in *Caspase-3* and -*9* fold expression (*p* < 0.001) when compared to control samples [31]. Li et al., (2018) intended to discover the effects and mechanism of miR-195 regulation in colon cancer. *WNT-3a* and *β-catenin* were downregulated in colon cancer cell lines. *β-catenin* protein level and cell viability analysis showed statistically significant values in the Student’s t test [32]. In our study, the cytotoxicity of MMBL was confirmed by the induction of apoptosis in MCF-7 cells through Annexin V/PI assays (*p* < 0.0001). Al-Malky et al., in 2019, did similar work on MCF-7 cells to evaluate the role of a natural product resveratrol (RSVL) on the sensitization of human breast cancer cells (MCF-7) to investigate the effect of Doxorubicin in an effort to minimize its actual dose and, thus, its side effects [33]. Taparia and Khanna., in 2016, worked on cocoa catechins and procyanidin extracted from *Theobroma cacao* L. and found their significant effect in the induction of apoptosis through Annexin V/PI assays [34]. Similar to our finding, β-sitosterol derived from *Acalypha wilkesiana* was tested in MCF-7 human breast cancer cell lines by utilizing resazurin assays and flow cytometry by Halimah et al. [35]. β-sitosterol inhibits MCF-7 breast cancer cell proliferation and displays dose-dependent apoptotic action. The findings of this study suggest that β-sitosterol could be used to treat cancer [35]. Hexadecanoic acid, an important bioactive compound present in our compound, is known to have antiapoptotic activity. Sangpairoj et al. (2022) found that a cytotoxic concentration of Hexadecanoic acid-enriched extract of *Halymenia durvillei* induced the apoptotic death of MDA-MB-231 cells via mitochondrial membrane dysfunction, the induction of apoptosis markers, and the increased expression of proteins related to DNA damage response [36]. One of the common compounds detected in MMBL is stigmasterol, which is a natural phytosterol component in the plant that has been shown to have an essential role in producing anti-inflammatory and anticancer activities. In 2022, AmeliMojarad and colleagues discovered that it reduced Bcl-2 and BCL-XL gene expression (* *p* < 0.05), induced apoptosis, and hindered cell proliferation in MCF-7 cell lines. Stigmasterol has been postulated as a viable medication in breast cancer treatment or as an adjuvant due to its ability to lower the expression of specific genes and increase apoptosis [37]. Our findings further demonstrated statistically substantial downregulation of *WNT-3a* and *β-catenin* by MMBL on MCF-7 cells in real-time PCR, as well as the considerable induction of apoptosis in the Annexin/PI test. Prasad et al. (2009) investigated curcumin, a substance isolated from Curcuma longa, and discovered a downregulation of *β-catenin* in breast cancer cell lines by MTT and Western blot analysis [38]. Flavonoids are polyphenolic compounds generated from plants that have antioxidative, anti-inflammatory, anti-viral, and anti-tumoral properties. Amado et al. (2014) used in vitro models as well as in vivo experiments in Xenopus embryos, a functional model of canonical *WNT* signaling research, to determine whether isoquercitrin influenced *WNT/β-catenin* signaling. Similar to our findings, they suggest that isoquercitrin has an inhibitory effect on *WNT/β-catenin*, with the flavonoid acting downstream of *β-catenin* translocation to the nuclei [39]. PEITC (phenethyl isothiocyanate) is an anticancer natural substance derived from cruciferous vegetables. Chen et al. (2018) studied the inhibitory effect of PEITC on colorectal CSCs as well as the underlying mechanisms. Given that the *WNT/β-catenin* route is important for CSC stemness, we investigated whether PEITC influenced *WNT/β-catenin* pathway activation. PEITC treatment significantly lowered phosphorylated GSK-3β and elevated GSK-3β, thus resulting in *β-catenin* (58%; *p* < 0.05) and its downstream target *c-Myc* (53%; *p* < 0.05) downregulation [40].

We used curcumin and resveratrol as reference drugs. Both drugs are two natural compounds that have been extensively studied for their potential therapeutic benefits, including anti-inflammatory, antioxidant, and anti-cancer effects. The MTT assay is a commonly used in vitro assay for evaluating the viability and proliferation of cells. Curcumin and resveratrol are often used as standard natural drugs in MTT assays because of their well-known properties and established pharmacological profiles. These compounds have been shown to affect cell viability and proliferation in a dose-dependent manner, making them useful as positive controls in MTT assays. Moreover, curcumin and resveratrol are readily available and relatively inexpensive, making them ideal for use in MTT assays. They also have low toxicity and are generally considered safe for use in vitro [41,42,43].

## 4. Materials and Methods

### 4.1. Materials

The following were purchased from Sigma-Aldrich (Dorset, UK): Dulbecco’s Modified Eagle Medium (DMEM), Penicillin/Streptomycin, MEM non-essential amino acids 100x, Trypsin-EDTA, L-glutamine. Foetal bovine serum (FBS) was from Gibco Life Technologies, Sao Paolo, Brazil. The protease and phosphatase inhibitors cocktail were from Roche, Indianapolis, IN, USA. The following antibodies were purchased from Abcam Biotechnology, Cambridge, UK: rabbit monoclonal [EPR26155-110-1] to *β-catenin*, rabbit monoclonal [EPR21889] to *WNT-3a*, rabbit monoclonal [E87] to *Caspase-3*, and rabbit polyclonal to GAPDH—the loading control. Curcumin, resveratrol, and Doxorubicin were purchased from Sigma-Aldrich, St. Louis, MO, USA.

#### 4.1.1. Plant Collection

*M. buxifolia* was collected from the Lower Dir area (GPS coordinates: 34°50′43.19 north latitude and 71°54′16.43 east longitude) of Pakistan. The specimen was collected in May 2021, which is the peak flowering season of *M. buxifolia*, which was later identified and authenticated by a taxonomist at the Department of Botany, Arid Agriculture at Rawalpindi University, Pakistan and was given the voucher number: PMAS-346. The aerial part of plant was washed three to four times under tap water to remove the dirt and later dried under shade for three weeks [44]. The dried plant leaves were pulverized into powdered form and kept in an airtight container for future use.

#### 4.1.2. Extract Preparation

Maceration method [44] was used to prepare the extract of *M. buxifolia* leaves. A total of 100 g of plant powder was taken and soaked in 300 mL of 80% methanol in a stoppered container and was placed on an orbital shaker at 120 rpm for 72 h at room temperature. The mixture was then filtered through Whatman paper no. 40, and the filtrate was pressed under reduced pressure using a rotary evaporator until dry extract was left behind and stored in a 4 °C refrigerator for further use. Simply, to obtain a crude methanol extract, the solution was filtered, followed by removing of solvent under reduced pressure. The extraction yield (%) was calculated as follows:

(1) $$\begin{array}{c} \mathrm{Extract}\;\mathrm{yield}\;\%=\frac{\mathrm{Weight}\;\mathrm{of}\;\mathrm{the}\;\mathrm{extract}\;\mathrm{after}\;\mathrm{evaporating}\;\mathrm{solvent}\;\mathrm{and}\;\mathrm{freeze}\;\mathrm{drying}\;}{\mathrm{Dry}\;\mathrm{weight}\;\mathrm{of}\;\mathrm{the}\;\mathrm{sample}}\times 100 \end{array}$$

### 4.2. Total Phenolic Contents and Total Flavonoid Contents

The total phenolic contents (TPC) of MMBL were determined [27] as gallic acid equivalents (mg gallic acid/g dried extract). Total flavonoid contents were determined [44] as rutin equivalents (mg rutin/g dried extract). Methanol was used as the blank. The evaluations were carried out in triplicate.

TPC/TFC = c. V/m(2)

where c is the sample concentration from the calibration curve (mg/mL), V is the volume (mL) of the solvent used for the extraction, and m represents the weight (g) of the dried sample used.

### 4.3. Characterization

#### 4.3.1. Fourier Transform Infrared Spectrophotometer (FTIR)

The sample was crushed with KBr (1:10), and the pellet was made with a hydraulic press. The pellet was then loaded inside the FTIR (Shimadzu, IR Affinity 1, Japan) with a 400 to 4000 cm^−1^ scanning array and at resolution of 4 cm [12].

#### 4.3.2. Gas Chromatography Mass Spectrometry

The volatile components of MMBL were investigated using a gas chromatography mass spectrometer (GCMS). The extract was prepared, and the GC used for analysis was outfitted with a column (fused silica, ZB-5MS) that was linked to an MS (JMS 600-H). The oven temperature for the column was initially set at 80 °C for 5 min, then increased to 150 °C at a 5 °C/min sequential speed, then to 250 °C at 10 °C/min for 10 min, and finally to 275 °C at 10 °C/min for 30 min. The temperatures of the front injector and detector were set to 250 °C and 260 °C, respectively. For injection, a split ratio of 80 eV was chosen. Helium was used as the carrier gas, with a flow rate of 1.7 mL/min [45]. The compounds identified in the MMBL via GCMS examination were validated by their retention time contrast with the spectral data stored in Wiley and NIST library [46].

### 4.4. Radical Scavanging Assays

#### 4.4.1. DPPH Assay

The DPPH foraging assay was used to determine scavenging activity of the free radicals. The 0.004% DPPH stock solution (absorbance 0.8–0.9 AU at 517 nm) was prepared in 100 mL methanol and kept at 20 °C for further use. Serial dilutions for standard and plant extracts were made in 1.5 mL Eppendorf tubes; 100 μL from each dilution was mixed with 900 μL DPPH solution and was set aside at room temperature in the dark for 30 min. The absorbance values were taken using spectrophotometry at 517 nm. The 50% scavenging of free radicals for each standard and plant extract was measured, and EC50 value was further evaluated.

#### 4.4.2. Total Antioxidant Capacity (TAC) Assay

TAC Assay determines total antioxidant capacity of MMBL which can be expressed as equivalent to standard i.e., mg of ascorbic acid/g of extract. A total of 2.5 mg stock solution of each extract was prepared in DMSO as a stock sample with five serial dilutions. A total of 100 μL of the sample was taken in 1.5 mL Eppendorf tubes and mixed with 1.0 mL of the reagent mixture (4 mM ammonium molybdate, 0.6 M sulfuric acid, and 28 mM Sodium Phosphate); the Eppendorfs were well capped with parafilm and incubated in a water bath at 95 °C for 90 min, and absorbance was measured at 765 nm using spectrophotometry. The total antioxidant capacity was measured using the standard curve [47].

### 4.5. Cell Culture and Cell Viability Analysis

The human breast cancer cell line MCF-7 (from ECACC) was used in this study with ethical approval number: No. IIUI/ORIC/Bioethics/110-81. The cells were grown under standard conditions of 37 °C in a humidified atmosphere with 5%, CO_2_-95% air in T75-cm^2^ flasks containing 15 mL DMEM supplemented with 1% (*v*/*v*) L-glutamine, 1% (*v*/*v*) penicillin/streptomycin, and 10% (*v*/*v*) FBS. The medium was accompanied with 1% (*v*/*v*) non-essential amino acids [48].

#### 4.5.1. MTT Assay

MTT assay was performed to determine the viability of the cells. The MCF-7 breast cancer cell lines were used at passage 3 stage with about 10,000 cells per well in a 96 well plate. Once the cells were seeded, they were left to be attached for 48 h inside the incubator. After that, the cells were dosed with DMEM containing different extract concentrations, including curcumin (10 mM stock) and resveratrol (50 mM stock), to compare with MMBL. The MTT assay was performed by adding 10 μL of MTT to each well containing cells and incubated for 3 h. Later, the media was removed, 150 μL DMSO was added to each well, cells were agitated on an orbital shaker (75 rpm) for 15 min, and absorbance was measured at 590 with a reference filter at 620 nm. All the experiments were repeated in triplicates.

#### 4.5.2. Acid Phosphatase Assay

The cells were seeded and dosed for the same number of hours as above. After 48 h of treatment, the media was removed, cells were washed twice with PBS to remove all the media, 100 μL of acid phosphatase buffer was added, and the plate was incubated for 1 h again. Later on, 50 μL of 0.25 M NaOH was added to halt the reaction. Absorbance was measured at 405 nm in the microplate reader [49].

### 4.6. Real-Time PCR Targeting WNT/β-Catenin Genes

#### 4.6.1. Selection of Doses

The doses were selected on the basis of cytotoxicity assays, and doses were adjusted around their IC 50 values. The MCF-7 cells were treated with two different doses of MMBL and processed further for expression studies through real-time PCR and Annexin V/PI assay.

#### 4.6.2. RNA Extraction Using TRIzol Method

Total RNA from treated MCF-7 cells was extracted with the TRIzol reagent. A total of 1 mL TRIzol (Thermo Fisher Scientific, Waltham, MA, USA) was added to 106 cells and kept at room temperature. After 5 min incubation, 200 μL chilled chloroform was added and centrifuged at 10,000× *g* for 20 min. The supernatant was discarded, 200 μL chilled isopropanol was added, and the solution was mixed and incubated on ice for 10 min. Quantification of extracted RNA was done using nanodrop and 1% agarose gel electrophoresis for quality assurance [50].

#### 4.6.3. Expression Profiling of *Caspases-1, -3, -7, -9*, *WNT-3a*, and *β-Catenin* Genes

Following RNA extraction, transcription to cDNA was performed using a cDNA synthesis kit (Vivantis cDSK01-050). The cDNA was used to govern the manifestation of *Caspase-1, -3, -7, -9, WNT-3a*, and *β-catenin* genes. Glyceraldehyde-3-phosphate dehydrogenase (*GAPDH*), one of the most commonly used housekeeping genes for comparisons of gene expression data, was used as housekeeping gene. Expression profiling was performed on MIC qPCR (BMS, Australia). All samples, including treated and untreated, were run in triplicate using SYBR green method, and relative expression was determined by using 2^−ΔΔCT^ method [51]. List of primers used is given in the supplementary data (Tables S3 and S4).

### 4.7. Apoptosis Detection by Annexin V and PI Staining

MCF-7 cells were treated with 100 g/mL of MMBL for 48 h. A total of 1 × 10^6^ cells were taken and 500 μL of 1% FBS plus 200 μL of Annexin V binding buffer were added. The cells were kept for 5 min at room temperature prior to centrifugation at 400 RCF for 5 min. The supernatant was discarded and palate was resuspended in 100 μL of Annexin V binding buffer by gently tapping. Formerly, 5 μL of Annexin V and 5 μL of propidium iodide were added, and cells were incubated in dark for 20 min. After washing, the cells were resuspended in 100 μL of PBS. The stained cells were shifted into Facs tubes and were acquired on FACScan flow cytometer (BD Biosciences, San Jose, CA, USA) using an argon laser at 488 nm. Data were analyzed on Cell Quest software [29].

### 4.8. Western Blot Analysis

Cells were lysed using RIPA buffer (50 mM Tris-HCl pH 7.4, 150 mM NaCl, 1% NP-40, 0.1% SDS, 2 mM EDTA) after 24 h of treatment with 100 g/mL of MMBL. The BCA test was used to determine protein concentration, and equivalent amounts were electrophoresed in SDS polyacrylamide gel before being transferred onto nitrocellulose membranes. Membranes were subsequently immunoblotted with monoclonal antibodies employed at 1:500–1000 dilutions as primary antibodies, while at a 1:2000 dilution as a secondary antibody. Chemiluminescence detecting kit was used to create the membranes (Santa Cruz Biotechnology, Santa Cruz, CA, USA). A Biorad ChemiDoc MP was used to photograph the membranes.

### 4.9. Statistical Analysis

The statistical analysis was done using one-way ANOVA and Tukey’s test to analyze the significant differences between the control and treated groups. A two-way ANOVA was used to analyze flow cytometry data. The values shown were considered statistically significant (*p* < 0.001).

## 5. Conclusions

*M. buxifolia* can be recommended as a probable natural source of antioxidants that is suitable for breast cancer treatment. The *M. buxifolia* persuaded the upregulation of proapoptotic marker genes (*Caspase-1, -3, -7* and *-9*) and the downregulation of the *WNT* pathway associated genes *WNT-3a* and *β-catenin*, which caused apoptotic cell death in human breast cancer cells. Thus, *M. buxifolia* could be used as a basis of novel lead structures in drug strategy to fight cancer. However, advanced assessment of its bioactive composites, along with antioxidant activities in animal models, must be performed. Hence, on the basis of further experimental validation, our results may aid in the active treatment of breast cancer in the future.

## Figures and Tables

**Table 1:** GCMS analysis of *Monotheca buxifolia*.

RT (min)	Compound Name	CAS No.	Abundance (%)	Mol. Formula	Mol. Weight (a.m.u.)	Compound Nature	Bioactivity	Reference
16.60	Heptadecanoic acid	000506-12-7	1.30	C_17_H_34_O_2_	270.5	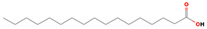	Antidiabetic	[13]
17.30	8,11-Octadecadienoic acid, meth.	056599-58-7	1.62	C_19_H_34_O_2_	294.5	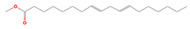	Antihypertensive, anticoagulant	[14]
17.58	Phytol	070928-44-8	2.46	C_20_H_40_O	296.5	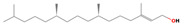	Antimicrobial, anti-inflammatory, cytotoxic, diuretic	[15]
18.25	9,12-Octadecadienoic acid (Z,Z)-	000060-33-3	19.54	C_18_H_32_O_2_	280.4	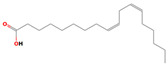	Hepatoprotective, antihistaminic, hypocholesterolemic, anti-eczemic	[16]
18.32	9,12,15-Octadecatrienoic acid,	000301-00-8	5.50	C_18_H_30_O_2_	278.4	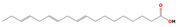	Antihistaminic, anticoronary, insectifuge, anti-eczemic, anti-acne	[17]
20.94	Linoleic acid ethyl ester	000544-35-4	1.22	C_20_H_36_O_2_	308.4986	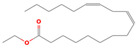	Hypocholesterolemic, hepatoprotective, insectifuge, anti-eczemic, anti-acne antihistaminic, anticoronary	[18]
23.78	1,2-Benzenedicarboxylic acid, m.	004376-20-9	6.17	C_8_H_6_O_4_	166.1308	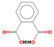	Antibacterial	[19]
32.12	D,.alpha.-Tocopherol	1000128-08	0.54	C_29_H_50_O_2_	430.7	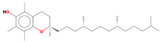	Antioxidant, anti-inflammatory, anti-cancer	[20]
33.93	Campesterol	000474-62-4	2.32	C_28_H_48_O	400.7	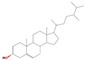	Cytotoxic, anti-inflammatory	[21]
34.66	Stigmasterol	000083-48-7	2.89	C_29_H_48_O	412.7	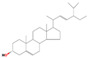	Antioxidant, antimicrobial, anti-cancer, anti-arthritic, anti-inflammatory, antigungal	[22]
36.23	Sitosterol	000083-47-	15.95	C_29_H_50_O	414.7	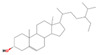	Anti-osteoarthritic, anti-cancer, anti-inflammatory, anti-neurological, antioxidant, and antimicrobial	[19]
15.84	n-Hexadecanoic acid	000057-10-3	6.68	C_16_H_32_O_2_	256.4	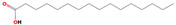	Cytotoxic, antioxidant	[20]
15.96	Hexadecanoic acid, ethyl ester	000628-97-7	3.45	C_18_H_36_O_2_	284.4772	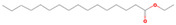	Antioxidant, hypocholesterolemic, nematicide, pesticide, lubricant, antiandrogenic, flavor, hemolytic 5-alpha reductase inhibitor	[21]

RT = retention time.

**Table 2:** Cytotoxicity analysis of MMBL along with standard natural drugs.

Sample	MTT IC_50_ μg/mL	APT IC_50_ μg/mL
MMBL	232 μg/mL	173 μg/mL
Curcumin	13.7 μg/mL	11.5 μg/mL
Resveratrol	15.1 μg/mL	9.2 μg/mL

MMBL: methanolic extract of *M. buxifolia* leaves; MTT: (3-(4,5-dimethylthazolk-2-yl)-2,5-diphenyl tetrazolium bromide) assay; APT: acid phosphatase assay.

**Table 3:** Summary of *Casp-1* and *Casp-3*-fold expression by real-time PCR for MMBL (*n* = 6).

Treatments/Samples	Targeting *Casp-1*		Targeting *Casp-3*	
	Mean of ∆∆CT	Fold Expression 2^−∆∆Cq^	Mean of ∆∆CT	Fold Expression 2^−∆∆Cq^
NTC	0.00 ± 0.06	1.00 ± 0.04	0.00 ± 0.25	1.01 ± 0.17
Doxorubicin 0.75 μg	−1.25 ± 0.02	2.38 ± 0.03	−1.78 ± 0.09	3.44 ± 0.22
MMBL 100 μg/mL	−1.05 ± 0.01	2.03 ± 0.15	−1.48 ± 0.02	2.79 ± 0.04
MMBL 300 μg/mL	−1.17 ± 0.09	2.16 ± 0.19	−1.61 ± 0.01	3.04 ± 0.03

*Casp-1*: *Caspase 1*; *Casp-3*: *Caspase 3*; MMBL: methanolic extract of *M. buxifolia* leaves.

## Data Availability

All data is presented in the manuscript.

## Supplementary Materials

The following supporting information can be downloaded at: https://www.mdpi.com/article/10.3390/plants12051147/s1, Figure S1: DPPH radical scavenging assay was performed using methanolic extract of *M. buxifolia* at varying concentrations (**A**); Phosphomolybdate assay was per-formed using different concentrations of methanolic extract of *M. buxifolia* (**B**); Total phenolic contents were evaluated using Gallic acid as a standard (**C**); and Flavonoid contents were evaluated using Rutin as a standard (**D**); Figure S2: FTIR analysis of *M. buxifolia*; Figure S3: GCMS analysis of *M. buxifolia*; Table S1: Summary of *Casp-7* and *Casp-9*-fold expression by Real time PCR for methanolic *M. buxifolia* extract (*n*= 6); Table S2: Summary of *WNT-3a* and *β-catenin* gene fold expression by Real time PCR for methanolic *M. buxifolia* extract (*n*= 6); Table S3: List of primers used in the study; Table S4: Optimized PCR conditions.
