# Supplementary material for: In Vitro Analysis of Cytotoxic Activities of Monotheca buxifolia Targeting WNT/β-Catenin Genes in Breast Cancer Cells
Source: Plants (Basel). 2023 Mar 2;12(5):1147. doi: 10.3390/plants12051147 (PMC10005423; doi:10.3390/plants12051147)
Supplement: Supplementary file 1 [file plants-12-01147-s001.zip › plants-2233619-supplementary.pdf]

plants

Article

In Vitro Analysis of Cytotoxic Activities of *Monotheca buxifolia* Targeting *WNT/β-Catenin* Genes in Breast Cancer Cells

Ambreen Sher [1], Sobia Tabassum [1,*], Heather Mann Wallace [2], Asifullah Khan [3], Asad Mustafa Karim [4], Sarah Gul [1] and Se Chan Kang [4,*]

[1] Department of Biological Sciences, Faculty of Basic and Applied Sciences, International Islamic University, Islamabad 44000, Pakistan
[2] Institute of Medical Sciences, University of Aberdeen, Aberdeen AB24 3FX, UK
[3] Department of Computer and Information Sciences, Pakistan Institute of Engineering and Applied Sciences (PIEAS), Islamabad 44000, Pakistan
[4] Graduate School of Biotechnology, Department of Oriental Medicine Biotechnology, Kyung Hee University, Yongin 17104, Republic of Korea
* Correspondence: sobia.tabasum@iiu.edu.pk (S.T.); sckang@khu.ac.kr (S.C.K.)

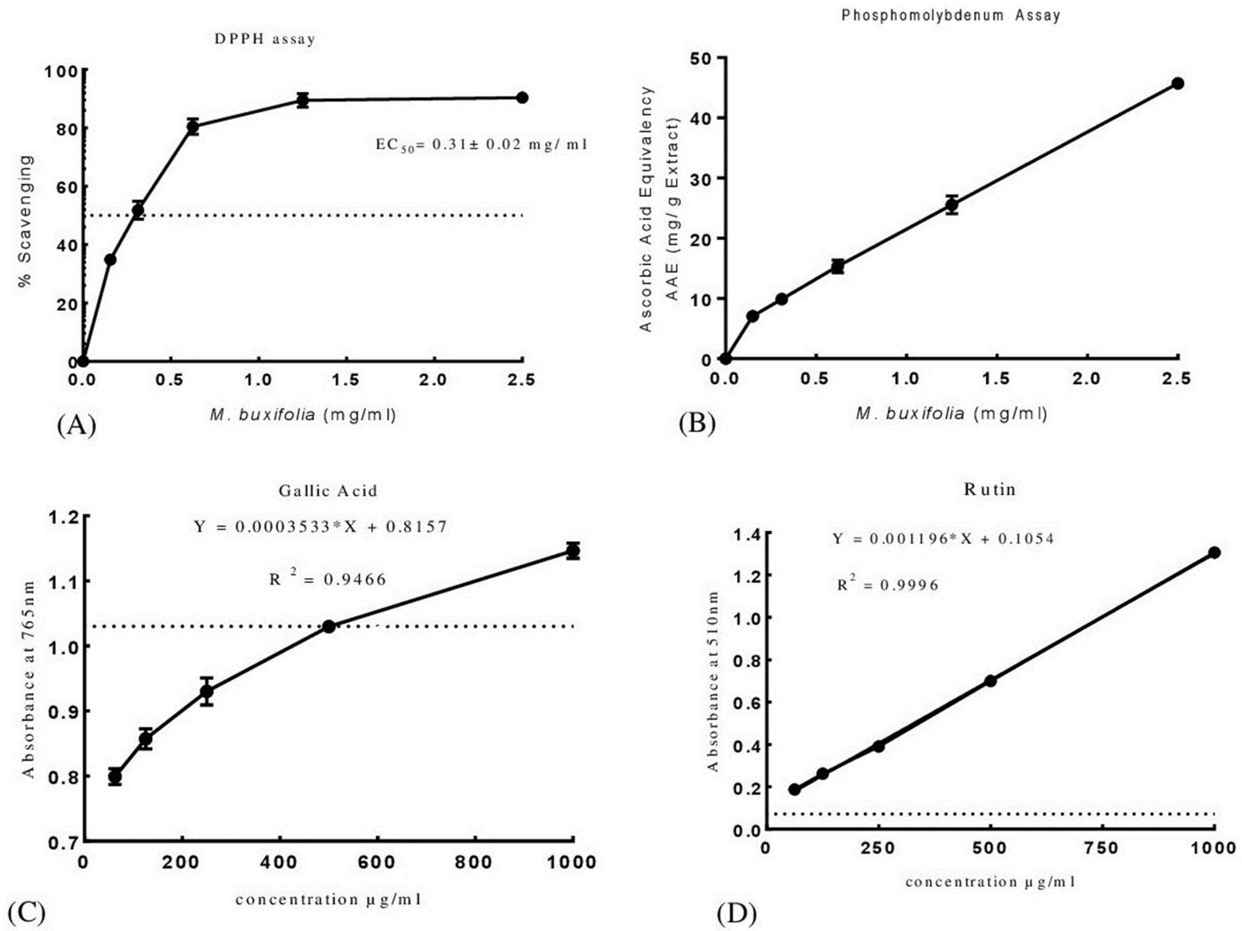

Figure S1. DPPH radical scavenging assay was performed using methanolic extract of M. buxifolia at varying concentrations **(A)**; Phosphomolybdate assay was performed using different concentrations of methanolic extract of M. buxifolia **(B);** Total phenolic contents were evaluated using Gallic acid as a standard **(C); and** Flavonoid contents were evaluated using Rutin as a standard **(D).** Each value represents a mean ± SD.

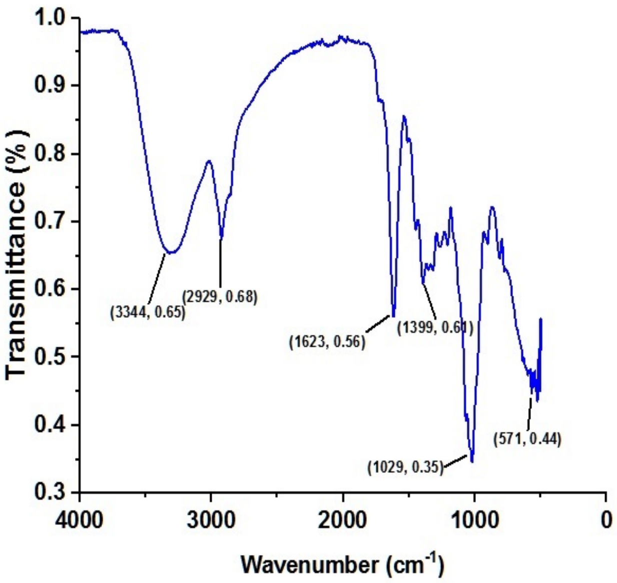

Figure S2. FTIR analysis of *M. buxifolia*.

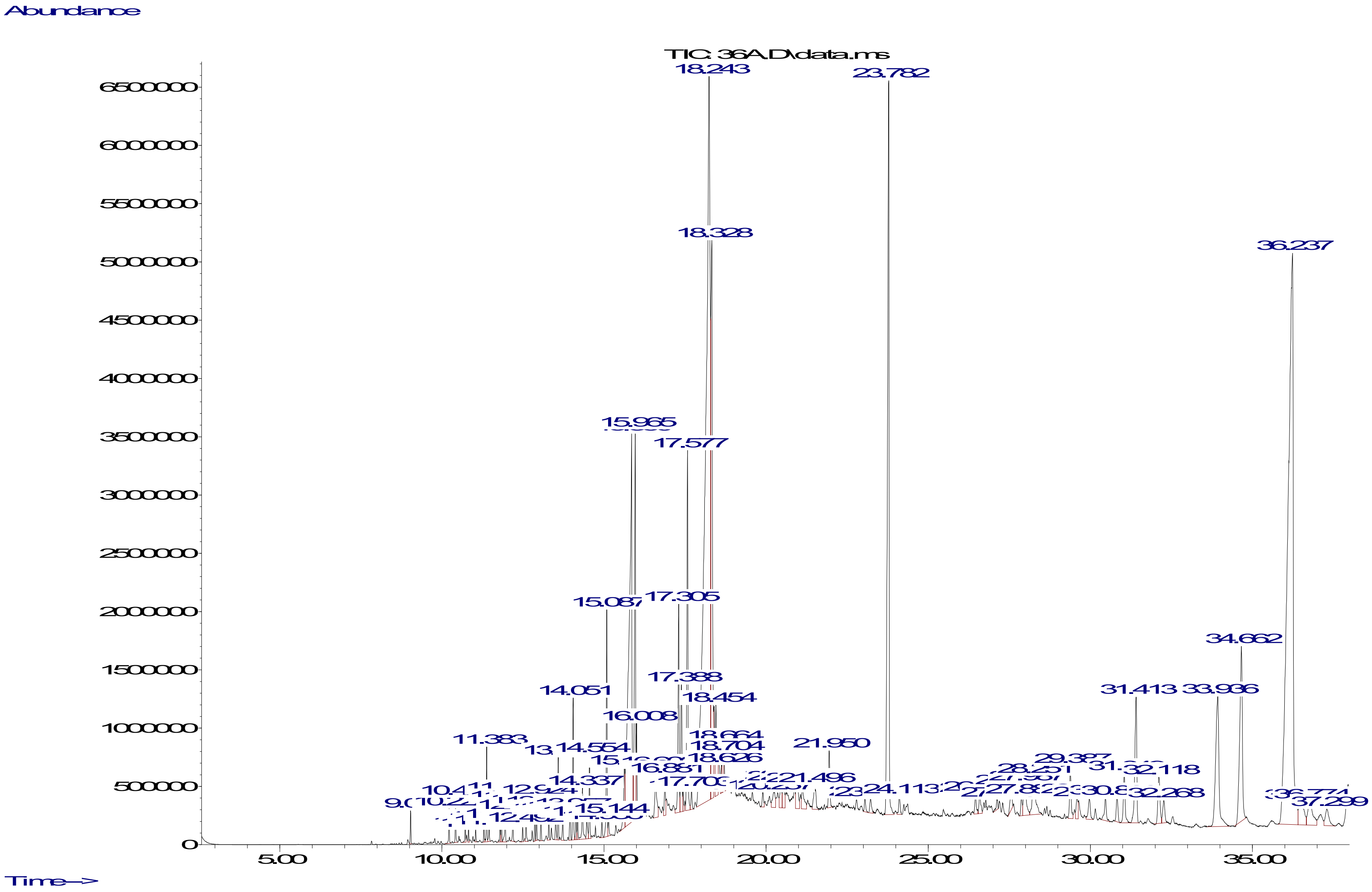

Figure S3. GCMS analysis of *M. buxifolia*.

Table S1. Summary of Casp- 7 and Casp-9-fold expression by Real time PCR for methanolic *M. buxifolia* extract (n= 6).

**Treatments/ samples**	**Targeting *Casp-7***		**Targeting *Casp-9***	
	**Mean of ΔΔ CT**	**Fold Expression**	**Mean of ΔΔ CT**	**Fold Expression**
NTC	0.00 ± 0.59	1.04 ± 0.41	0.00 ± 0.15	1.00 ± 0.01
Doxorubicin 0.75 μg	-1.71 ± 0.08	3.26 ± 0.05	-1.82 ± 0.20	3.52 ± 0.48
M. buxifolia 100 μg/ ml	-1.19 ± 0.04	2.28 ± 0.06	-1.10 ± 0.08	2.14 ± 0.13
M. buxifolia 300 μg/ ml	-1.45 ± 0.04	2.72 ± 0.07	-1.40 ± 0.01	2.64 ± 0.01

Table S2. Summary of Wnt- 3a and β- catenin gene fold expression by Real time PCR for methanolic *M. buxifolia* extract (n= 6).

**Treatments/ samples**	**Targeting *Wnt- 3a* gene**		**Targeting *β- catenin* gene**	
	**Mean of ΔΔ CT**	**Fold Expression**	**Mean of ΔΔ CT**	**Fold Expression**
NTC	0.00 ± 0.41	1.02 ± 0.28	0.00 ± 0.06	1.00 ± 0.04
Doxorubicin 0.75 μg	2.77 ± 0.09	0.14 ± 0.007	1.09 ± 0.09	0.47 ± 0.02
M. buxifolia 100 μg/ ml	2.36 ± 0.11	0.20 ± 0.01	0.55 ± 0.04	0.68 ± 0.01
M. buxifolia 300 μg/ ml	2.25 ± 0.02	0.21 ± 0.001	0.66 ± 0.04	0.63 ± 0.01

Table S3. List of primers used in the study.

Sr. No	Primer Name	Sequence	Product size (bp)
1	Caspase-1_F	TACCTCTTCCCAGGACATTA	110
2	Caspase-1_R	ATGGACTTTCAGTACCCTTTC	
3	Caspase-3_F	GCCTGTAACTTGAGAGTAGATG	114
4	Caspase-3_R	GTATGGAGAAATGGGCTGTAG	
5	Caspase-7_F	TACCTTGGTCTCACCTATCC	143
6	Caspase-7_R	CCAGGCTTACATCCATTTCT	
7	Caspase-9_F	GAGACAGAGTTATGCCATGTTA	136
8	Caspase-9_R	GGCCCTAAGAACCAGAAATAG	
9	Wnt- 3a_F	GGCACTAGGTAGGCTTCTA	142
10	Wnt- 3a _R	GAAGCCTCATCCACCATAAA	
11	β Catenin_F	GCTTTAGGACTTCACCTGAC	105
12	β Catenin_R	CTTCCATCCCTTCCTGTTTAG	
13	GAPDH_F	GTATGACAACAGCCTCAAGAT	196
14	GAPDH_R	GTCCTTCCACGATACCAAAG	

Table S4. Optimized PCR conditions.

**Steps**	**Sub-cycles**	**Conditions**	**PCR cycles**
**Initial Denaturation**		95 °C, 12 min	1
**PCR Cycles**	Denaturation	95 °C, 15 sec	40
	Primer annealing	61°C, 20 sec	
	Primer extension	72 °C, 20 sec	
**Hold**		95 °C, 15 sec	
